# The effect of omega-3 polyunsaturated fatty acids on short-chain fatty acid production and the gut microbiome in an *in vitro* colonic fermentation model

**DOI:** 10.1017/gmb.2025.10016

**Published:** 2026-01-06

**Authors:** Joanna Aldoori, Suparna Mitra, Alexander Davie, Giles J. Toogood, Christine Edwards, Mark A. Hull

**Affiliations:** 1 Leeds Institute of Medical Research, University of Leeds, UK; 2 Hepatobiliary Surgery, Leeds Teaching Hospitals NHS Trust, UK; 3 School of Medicine, Dentistry and Nursing, College of Medical, Veterinary and Life Sciences, University of Glasgow, UK

**Keywords:** omega-3 polyunsaturated fatty acids, inulin, short-chain fatty acids

## Abstract

Oral administration of omega-3 polyunsaturated fatty acids (PUFAs) to rodents and humans is associated with an increase in gut bacteria that are predicted to synthesise short-chain fatty acids (SCFAs). We tested the hypothesis that physiological levels of omega-3 PUFAs in the distal intestinal lumen (1–50 μg/mL) are associated with increased SCFA synthesis in an *in vitro* fermentation model using faecal slurry from 10 healthy participants (mean age 30 years), with and without exogenous dietary fibres. SCFAs were measured by gas chromatography-flame ionisation detection (*n* = 10), and changes in bacterial composition were analysed by shotgun metagenomic sequencing (*n* = 6). In the presence of omega-3 PUFAs, there was a mean 9.3% (no inulin; *P* = 0.03) and 19.3% (+ 0.01 mg/mL inulin; *P* = 0.01) increase in total SCFA concentration at 24 h compared with paired control fermentations. Omega-3 PUFAs had a limited effect on the fermentation model microbiome in the absence of inulin. However, omega-3 PUFAs (50 μg/mL) were associated with increased abundance of *Bifidobacteriaceae* compared with paired control fermentations, if inulin (0.01 mg/mL) was present. Prebiotic activity of omega-3 PUFAs drives SCFA synthesis in an *in vitro* colonic fermentation model and is augmented by the soluble fibre inulin.

## Introduction

Effective prevention strategies are required for colorectal cancer (CRC), which continues to represent a huge global health burden, despite major advances in diagnosis and cancer treatment (Murphy & Zaki, 2024). Therapeutic (chemo-) prevention of CRC with drugs or nutrients is a promising strategy, in combination with screening and surveillance programmes (Ruan et al., 2025). Omega-3 polyunsaturated fatty acids (PUFAs), including C20:5*n*-3 eicosapentaenoic acid (EPA) and C22:6*n*-3 docosahexaenoic acid (DHA), are found naturally in high quantities in oily fish and are also licensed for treatment of resistant hypertriglyceridaemia and secondary cardiovascular risk reduction as high-dose, high-purity formulations (Sherratt et al., 2024). There is epidemiological and randomised controlled trial (RCT) evidence that long-chain omega-3 PUFAs have anti-CRC activity (Aldoori et al., 2022). However, the mechanism(s) underlying the anti-neoplastic activity of omega-3 PUFAs in humans remain unclear (Volpato & Hull, 2018). Historically, mechanistic research into anti-CRC activity of omega-3 PUFAs has been largely restricted to *in vitro* cell and rodent models that focused on direct activity of omega-3 PUFAs on cancer cells (Volpato & Hull, 2018). The strongest line of evidence currently supports the notion that omega-3 PUFAs (particularly EPA) act, at least partly, via inhibition of cyclooxygenase-dependent prostaglandin (PG) E_2_ signalling, which is upregulated in CRC cells and drives pro-tumorigenic behaviours, including cell proliferation, resistance to apoptosis, enhanced motility, and angiogenesis (Wang et al., 2021). This is supported by evidence from RCTs of EPA, in which a reduction in levels of the stable urinary metabolite of PGE_2_ PGE-M was observed during omega-3 PUFA treatment (Sun et al., 2024). Alternatively, omega-3 PUFAs may also have indirect anti-cancer activity via augmentation of the host anti-tumour response (Westheim et al., 2022), the importance of which is now recognised for prevention and treatment of several solid cancers (Wang et al., 2021). This could also occur via inhibition of paracrine PGE_2_ signalling between tumour cells and stromal immune cell populations, leading to de-repression of the immune response to cancer cells by inhibition of suppressor T cells and myeloid-derived suppressor cells (Wang et al., 2021). An alternative hypothesis is that omega-3 PUFAs modulate the host anti-tumour response via changes to the intestinal microbiome, leading to increased synthesis of short-chain fatty acids (SCFAs), including C2 acetic acid, C3 butyric acid, and C4 propionic acid (where C denotes the number of carbon atoms in each SCFA), which have been demonstrated to modulate anti-tumour immune responses, in addition to direct pro-apoptotic activity on colonocytes (Westheim et al., 2022; Mann et al., 2024).

We have reported that oral administration of high-dose (4 g daily) mixed omega-3 PUFAs (1:1 EPA:DHA) altered the faecal microbiome of healthy, middle-aged adults, with an increase in abundance of several taxa linked to SCFA production, such as *Lactobacillus* and *Roseburia* (Watson et al., 2018). This is consistent with data from several independent animal studies (Caesar et al., 2015; Qiu et al., 2024). However, it has not been confirmed that oral administration of purified omega-3 PUFAs is associated with increased intestinal SCFA production in humans, although one study has reported a near-significant increase in serum C4 butyrate levels in healthy adults after dosing with 500 mg mixed omega-3 PUFAs daily for 6 weeks (Vijay et al., 2021).

In addition, we have demonstrated that EPA and DHA are present in the distal small intestine at concentrations up to 200 μg/mL after oral dosing with 4 g mixed omega-3 PUFAs (1:1 EPA:DHA) daily for 4 weeks (Nana et al., 2021). Therefore, we investigated whether exposure to physiologically relevant concentrations of omega-3 PUFAs is associated with increased synthesis of SCFAs by human faecal microbiota using a static *in vitro* human faecal fermentation model. An extension of the hypothesis that omega-3 PUFA treatment leads to increased colonic SCFA production is that dietary fibre would augment the activity of omega-3 PUFAs on luminal SCFA production. Therefore, we also tested whether soluble and insoluble dietary fibres interacted with omega-3 PUFAs in the *in vitro* fermentation model.

## Methods

Approval for the study was obtained from the University of Glasgow College of Medical, Veterinary and Life Sciences Ethics Committee (200210044).

### Study participants

Participants (P) were invited to take part in the study through poster advertisements on the University of Glasgow campus and using the University of Glasgow online social network service. Individuals were invited to join the study if they did not fulfil any of the following exclusion criteria: vegetarian or vegan diet; active pregnancy or aiming to become pregnant; regular (≥3 times per week) fish oil supplement (including cod liver oil) use; non-steroidal anti-inflammatory drug, laxative, or oral antibiotic use in the past 3 months; a diagnosis of inflammatory bowel disease or irritable bowel syndrome; previous intestinal resection or cholecystectomy, or active tobacco smoker. Written informed consent was obtained from all participants.

### Faecal collection and preparation

All participants were asked to avoid eating fish or other seafood products for 2 days before faecal collection. A stool sample was collected from each participant using an in-house collection kit that contained an AnaeroGen™ 3.5 L Sachet (Oxoid Ltd, Basingstoke, UK). Samples were processed within 2 h of collection. A faecal slurry (32% weight/weight) was prepared by homogenising the faecal sample for 1 min using a hand blender (Bosch MSM6300GB) and then adding 48 g of faeces into 150 mL of nitrogen-purged, oxygen-free sodium phosphate buffer (pH 7.0, 37 °C) before vortexing for 3 min. The faecal slurry was strained through nylon mesh to remove particulate matter before use in fermentation reactions. A 1 mL aliquot of faecal slurry was immediately placed at -80 °C for subsequent DNA extraction.

### Static in vitro fermentation reactions

Five millilitres of faecal slurry were added to 45 mL fermentation medium pH 7.0, containing 0.225% (w/v) tryptone, 0.01% (w/v) porcine stomach mucin, 0.0076% (w/v) porcine bile acids, and 0.0001% (v/v) resazurin in a 100 mL fermentation bottle sealed with a self-sealing septum and gas-tight crimp top, as described (Havlik et al., 2020). Each bottle was degassed using oxygen-free nitrogen for 2 min before the addition of the test intervention(s). Fermentations were kept at 37 °C in a shaking water bath at 60 strokes per minute for 24 h.

Each fermentation reaction was sampled by needle and syringe at baseline, 8 h, and 24 h for SCFA and omega-3 PUFA measurement (1 mL into 333 μL 1 M NaOH) and DNA extraction (1 mL taken at 24 h). All samples were stored at -80 °C. The pH of the fermentation reaction was measured in a 1 mL aliquot at baseline, 8 h, and 24 h using a 7020 pH meter (Electronic Instruments Ltd, Chertsey, Surrey).

### Omega-3 PUFA and dietary fibres

The omega-3 PUFA formulation (mixed [1:1] EPA and DHA triglycerides) was the same as that used in our previous clinical studies (Watson et al., 2018; Nana et al., 2021). Pure omega-3 PUFA oil was aspirated fresh from a soft-gel capsule for each experiment and added directly to the fermentation reaction at baseline to make working concentrations of 1, 25, and 50 μg/mL mixed omega-3 PUFAs, which mirrored the concentration range observed in human ileostomy fluid after oral administration of 4 g EPA and DHA triglycerides daily (Nana et al., 2021).

The soluble non-viscous fibre inulin (0.01 and 0.02 mg/mL; Orafti® GR [92% inulin; degree of polymerisation >10], Beneo GmbH, Mannheim, Germany), the soluble viscous fibre pectin (from apple; 0.01 mg/mL; Merck Life Sciences, Haverhill, UK), and the insoluble fibre wheat bran (0.01 mg/mL; food quality, Holland and Barrett, Nuneaton, UK) were added individually to separate fermentation reactions at baseline. The concentrations correspond to a bolus intake of ~6 g of fibre diluted in 300 mL of colonic content (Havlik et al., 2020), with the amount of fibre in each fermentation reaction being roughly equivalent to what would be expected after a meal (Harris et al., 2017; Thomson et al., 2021).

### SCFA extraction and analysis

SCFAs were measured by gas chromatography-flame ionisation detection (Agilent 7820A, Santa Clara, CA, USA) of acidified ether extracts of fermentation fluid, using 2-ethyl butyric acid as the internal standard, as described (Mansoorian et al., 2019). The following authentic SCFAs in 2 M NaOH were used: acetic acid (C2), propionic acid (C3), butyric acid (C4), isobutyric acid (iC4), valeric acid (C5), isovaleric acid (iC5), caproic acid (C6), isocaproic acid (iC6), enanthic acid (C7), and caprylic acid (C8). A more detailed description is available in Supplementary Methods.

### Liquid chromatography-mass spectrometry measurement of long-chain fatty acids

The levels of omega-3 PUFAs EPA and DHA in fermentation fluid at 8 and 24 h were measured by liquid chromatography–tandem mass spectrometry of lipid extracts, as described (Volpato et al., 2017). EPA and DHA levels were calculated from the ratio of the peak area to the internal standard (deuterated alpha-linolenic acid) peak used for quantification of the panel of nine fatty acids (Volpato et al., 2017). Data are expressed as the percentage of the baseline concentration of each omega-3 PUFA (50 μg/mL) at 8 and 24 h. A more detailed description is available in Supplementary Methods.

### Shotgun metagenomic analysis

DNA was extracted from fermentation fluid using a Qiagen QIAamp® PowerFecal® Pro DNA kit (Manchester, UK). Library preparation was carried out by the University of Leeds Genomics Facility using the New England Biolabs NEXT® Ultra™ DNA Library Prep Kit for Illumina® (Hitchin, UK) before shotgun metagenomic sequencing with an Illumina NextSq 2000 system (Cambridge, UK).

Low-quality reads were removed from FASTQ data using the Cutadapt tool (parameters -q 10, -m 30). Trimmed data had a mean of 35.9 million reads (Supplementary Data). Total reads were mapped onto the National Center for Biotechnology Information (NCBI) nr database (accessed June 2023). A mean of 34.9 million reads was assigned to NCBI-nr, although a few samples had a lower proportion of assigned reads (Supplementary Data). Therefore, we normalised the data to the sample with the lowest number of assigned reads (30,469,324). Taxonomic analysis at the Family- and Species-level was performed using MetaPhlAn 4.0. and *MEGAN* (MEtaGenome ANalyzer) 6 Ultimate was used to perform principal coordinate analysis (PCoA) based on Bray–Curtis dissimilarity. Functional profiling was performed using Kyoto Encyclopedia of Genes and Genomes (KEGG) release 112.1 linked to MEGAN6 Ultimate.

### Statistical analysis

SCFA levels are expressed as the mean and standard deviation (SD) of fermentations from all participants. The total SCFA level (mmol/L) was calculated as the sum of C2 acetate, C3 propionate, and C4 butyrate concentrations at baseline, 8, and 24 h. Levels of iC4 isobutyrate, C5 valerate, iC5 isovalerate, C6 caproate, iC6 isocaproate, C7 enanthate, and C8 caprylate were very low and are not reported. Preliminary experiments on duplicate fermentations from the same donor confirmed low intra-individual variability, so each experimental condition was limited to a single fermentation for each participant in order to minimise experimental size and cost for each set of fermentations per participant (Supplementary Figure 1A). Fermentations using faecal samples from the same donor taken 1 week apart confirmed the consistency of SCFA production in independent duplicate samples (Supplementary Figure 1B).

Changes in SCFA levels in the presence of omega-3 PUFAs and/or dietary fibres were expressed as the percentage change from the control (no omega-3 PUFAs) value. The paired *t*-test was used to compare absolute SCFA levels and the percentage difference between fermentation reactions. The decrease in omega-3 PUFA level from the baseline concentration at 8 and 24 h was tested by the Mann–Whitney *U* test. Comparison of the Shannon–Weaver diversity index of *in vitro* fermentations in the absence or presence of inulin was performed using the Mann–Whitney *U* test. Pairwise comparisons of bacterial abundance in the absence or presence of omega-3 PUFAs were performed using the Wilcoxon signed-rank test.

A *P*-value of <0.05 was considered statistically significant. All analyses were performed using R Studio version 4.1.2 or SPSS version 29.

### Sample size calculation

The overall number of participants required to generate sufficient statistical power to test the hypothesis that omega-3 PUFAs drive SCFA production in the *in vitro* fermentation model was determined using data from the first three participants. In the presence of the highest concentration of omega-3 PUFAs (50 μg/mL) and 0.01 mg/mL inulin, there was a mean 22% (SD 20%) increase in the total SCFA level at 24 h compared to the inulin-only reaction. Therefore, we calculated that a minimum of 10 participants would be required to detect a 20% increase in the primary outcome of total SCFA concentration, in the presence of omega-3 PUFAs, with 80% power at a two-tailed significance level of 0.05.

## Results

### Participant characteristics

Faecal samples were obtained from 10 healthy participants (5 male and 5 female) aged between 24 and 42 years (mean 30 [SD 5] years), who had a mean body mass index of 22.9 (SD 3.2) kg/m^2^. Seven participants were White British, two were Arab, and one was of South Asian ethnicity.

### The effect of omega-3 PUFAs on SCFA production during in vitro fermentation

**Figure 1.**
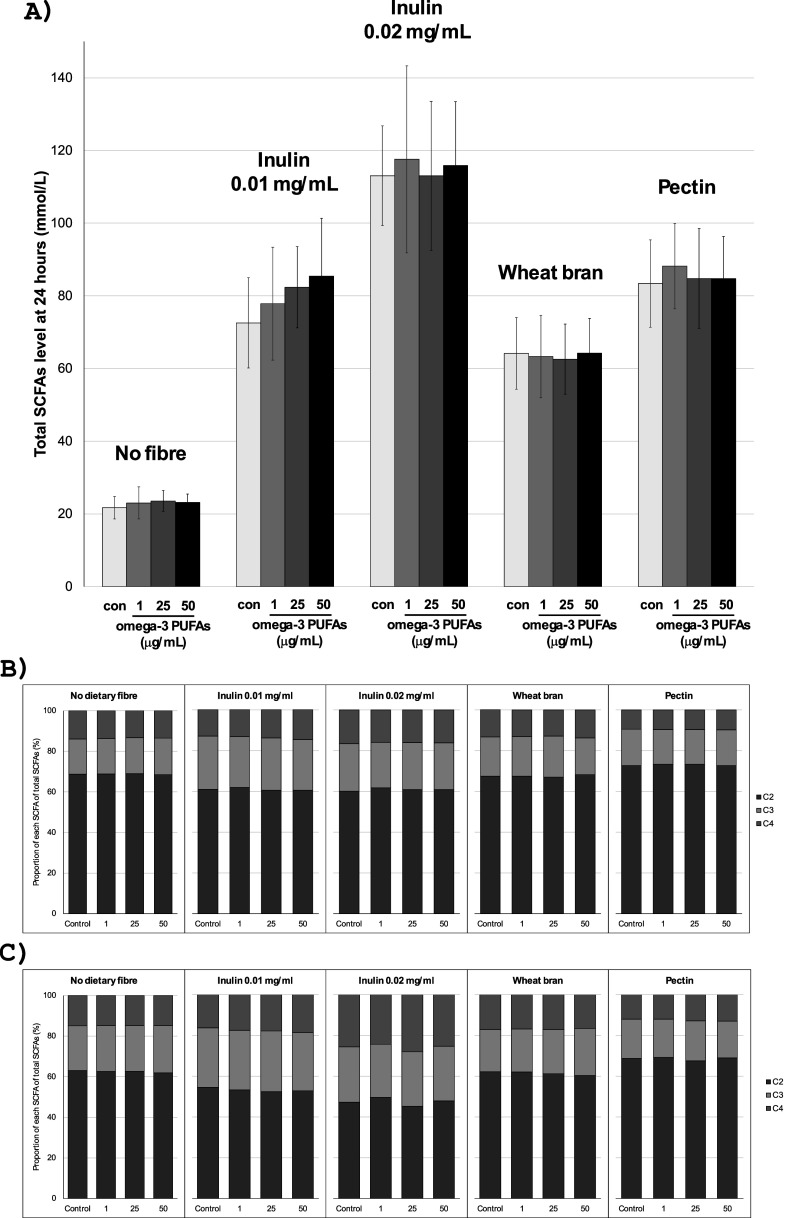
SCFA profiles in *in vitro* fermentation reactions in the presence of different dietary fibres. *Note*: (A) Total SCFA levels after 24-h incubation with no fibre, inulin (0.01 or 0.02 mg/mL), wheat bran (0.01 mg/mL), or pectin (0.01 mg/mL), in the absence (con) or presence of mixed omega-3 PUFAs. Data are the mean (column) and standard deviation (error bar) of data from 10 participants, except for the pectin values, which represent data from 9 participants, due to the small size of the faecal sample from one participant. (B) and (C) The proportion of C2 acetate, C3 propionate, and C4 butyrate of total SCFAs in *in vitro* fermentations with different dietary fibres at 8 h (B) and 24 h (C) according to the absence (control) or presence of exogenous omega-3 PUFAs (1–50 μg/mL).

In the absence of exogenous omega-3 PUFAs or dietary fibre, there was a <0.1 pH unit decrease in pH and a 6 mmol/L increase in SCFA levels over time during the 24-h *in vitro* fermentation period (Tables 1 and 2). The presence of mixed omega-3 PUFAs, in the absence of added fibre, was associated with a small, time-dependent degree of acidification and an increase in SCFA concentrations compared with control fermentations, which reached statistical significance at 24 h (Tables 1 and 2). SCFA profiles in fermentation reactions for each participant are presented in Supplementary Figure 2. The largest increase in SCFA levels was observed in the presence of 25 μg/mL omega-3 PUFAs, with a mean increase of 1.87 mmol/L (9.3%) in the total SCFA level (23.57 [SD 2.88] mmol/L) compared with control (21.70 [3.07] mmol/L; *P* = 0.03; Table 2) at 24 h. The relationship between omega-3 PUFA concentration and SCFA production was nonlinear, with a numerically smaller effect on SCFA levels during *in vitro* fermentation in the presence of 50 μg/mL omega-3 PUFAs compared with 25 μg/mL omega-3 PUFAs (Table 2 and Figure 1A). The increase in total SCFA levels in the presence of omega-3 PUFAs included an increase in each of the major SCFAs, acetate, propionate, and butyrate (Table 2).

**Table 1.** pH of *in vitro* fermentation reactions in the presence of omega-3 PUFAs

	Baseline	8 h	***P* ^2^**	24 h	***P* ^2^**
No omega–3 PUFAs	7.47 (0.07)^1^	7.40 (0.09)	0.01	7.40 (0.12)	0.04
Omega–3 PUFAs 1 μg/mL	7.48 (0.06)	7.39 (0.13)	0.01	7.32 (0.10)	<0.001
Omega–3 PUFAs 25 μg/mL	7.47 (0.07)	7.43 (0.12)	0.17	7.33 (0.08)	<0.001
Omega–3 PUFAs 50 μg/mL	7.46 (0.07)	7.41 (0.12)	0.05	7.33 (0.11)	0.002

PUFAs, polyunsaturated fatty acids.

1 Mean (standard deviation) pH value for *n* = 10 participants.

2 Paired *t*-test compared with the baseline value.

**Table 2.** Short-chain fatty acid levels in *in vitro* fermentation reactions over time in the presence of omega-3 PUFAs

Time	Experimental condition	Acetate (C2)^1^	Propionate (C3)	Butyrate (C4)	Total SCFAs	*P* ^2^	% change from control^3^	*P* ^2^
8 h	No omega–3 PUFAs	10.72 (2.34)	2.69 (0.61)	2.26 (0.86)	15.67 (3.55)	–	–	–
	Omega–3 PUFAs 1 μg/mL	11.31 (2.76)	2.87 (0.79)	2.34 (0.90)	16.53 (4.19)	0.17	4.9 (12.6)	0.25
	Omega–3 PUFAs 25 μg/mL	11.43 (2.35)	2.60 (0.68)	2.28 (0.86)	16.66 (3.64)	0.13	7.0 (13.1)	0.13
	Omega–3 PUFAs 50 μg/mL	10.60 (2.56)	2.78 (0.71)	2.18 (0.88)	15.57 (3.92)	0.87	−0.9 (11.8)	0.81
24 h	No omega–3 PUFAs	13.73 (2.67)	4.76 (0.94)	3.20 (0.63)	21.70 (3.07)	–	–	–
	Omega–3 PUFAs 1 μg/mL	14.37 (3.00)	5.25 (1.36)	3.39 (0.75)	23.01 (4.41)	0.24	6.2 (15.7)	0.25
	Omega–3 PUFAs 25 μg/mL	14.80 (2.42)	5.32 (1.02)	3.46 (0.85)	23.57 (2.88)	0.03	9.3 (10.6)	0.02
	Omega–3 PUFAs 50 μg/mL	14.39 (2.09)	5.42 (1.06)	3.37 (0.46)	23.18 (2.31)	0.03	7.6 (8.2)	0.02

C2, acetate; C3, propionate; C4, butyrate; PUFAs, polyunsaturated fatty acids; SCFAs, short-chain fatty acids.

1 Mean (standard deviation) SCFA level (mmol/L) for *n* = 10 participants.

2 Paired *t*-test comparing total SCFA level or % change with control (no omega-3 PUFAs).

3 Mean (standard deviation) % change of total SCFA level from the control (no omega-3 PUFAs) value.

### The effect of omega-3 PUFAs on SCFA production during in vitro fermentation with inulin

The addition of inulin to *in vitro* fermentation reactions was associated with marked inulin concentration-dependent acidification and a large increase in SCFA production compared with fermentations in the absence of exogenous inulin (Tables 3 and 4 and Figure 1A). SCFA profiles in fermentation reactions in the presence of 0.01 and 0.02 mg/mL inulin for each participant are presented in Supplementary Figures 3 and 4. There was clear concentration dependency with greater acidification and higher concentrations of all three SCFAs at both time points in the presence of 0.02 mg/mL inulin compared with 0.01 mg/mL inulin (Tables 3 and 4 and Figure 1A). Inulin substrate induced an increase in all three major SCFAs, with relative latency in the increase in butyrate levels, which was most prominent after 24 h, compared with acetate levels that often plateaued after the 8-h time point (Table 4 and Supplementary Figures 3 and 4).

**Table 3.** pH of *in vitro* fermentation reactions in the presence of omega-3 PUFAs and inulin

		Baseline	8 h	*P* ^2^	24 h	*P* ^2^
Inulin 0.01 mg/mL	No omega–3 PUFAs	7.47 (0.07)^1^	6.01 (0.38)	<0.001	5.54 (0.44)	<0.001
	Omega–3 PUFAs 1 μg/mL	7.47 (0.07)	5.96 (0.47)	<0.001	5.55 (0.39)	<0.001
	Omega–3 PUFAs 25 μg/mL	7.47 (0.07)	5.85 (0.33)	<0.001	5.41 (0.34)	<0.001
	Omega–3 PUFAs 50 μg/mL	7.46 (0.05)	5.90 (0.41)	<0.001	5.43 (0.39)	<0.001
Inulin 0.02 mg/mL	No omega–3 PUFAs	7.47 (0.07)	5.08 (0.33)	<0.001	4.74 (0.27)	<0.001
	Omega–3 PUFAs 1 μg/mL	7.46 (0.07)	5.11 (0.26)	<0.001	4.70 (0.19)	<0.001
	Omega–3 PUFAs 25 μg/mL	7.47 (0.07)	5.12 (0.30)	<0.001	4.86 (0.39)	<0.001
	)mega–3 PUFAs 50 μg/mL	7.46 (0.05)	5.12 (0.32)	<0.001	4.75 (0.20)	<0.001

PUFAs, polyunsaturated fatty acids.

1 Mean (standard deviation) pH value for *n* = 10 participants.

2 Paired *t*-test compared with the baseline value.

**Table 4.** Short-chain fatty acid levels in *in vitro* fermentation reactions over time in the presence of omega-3 PUFAs and inulin.

Inulin 0.01 mg/mL	Time	Experimental condition	Acetate (C2)^1^	Propionate (C3)	Butyrate (C4)	Total SCFAs	*P*^2^	% change from control^3^	*P*^2^
	8 h	No omega–3 PUFAs	32.48 (10.21)	14.27 (7.74)	7.22 (3.01)	53.96 (14.08)	–	–	–
		Omega–3 PUFAs 1 μg/mL	33.11 (10.77)	13.93 (7.86)	7.47 (5.10)	54.50 (16.38)	0.85	0.1 (15.7)	0.98
		Omega–3 PUFAs 25 μg/mL	36.72 (12.32)	15.72 (8.05)	8.88 (4.20)	61.33 (15.55)	0.03	14.5 (17.8)	0.03
		Omega–3 PUFAs 50 μg/mL	35.30 (12.17)	14.43 (7.16)	9.09 (4.60)	58.81 (15.47)	0.16	9.9 (15.5)	0.01
	24 h	No omega–3 PUFAs	39.92 (10.45)	20.88 (7.33)	11.77 (3.08)	72.57 (12.43)	–	–	–
		Omega–3 PUFAs 1 μg/mL	41.70 (10.70)	22.03 (6.14)	14.10 (6.74)	77.83 (15.51)	0.28	8.4 (21.1)	0.24
		Omega–3 PUFAs 25 μg/mL	43.83 (12.62)	23.70 (7.14)	14.83 (4.78)	82.36 (11.18)	0.02	15.3 (17.6)	0.02
		Omega–3 PUFAs 50 μg/mL	46.26 (17.41)	23.38 (8.10)	15.82 (5.30)	85.46 (15.87)	0.02	19.3 (20.3)	0.01

Inulin 0.02 mg/mL	Time	Experimental condition	Acetate (C2)^1^	Propionate (C3)	Butyrate (C4)	Total SCFAs	*P* ^2^	% change from control^3^	*P* ^2^
	8 h	No omega–3 PUFAs	38.74 (19.08)	16.29 (10.85)	12.77 (7.36)	67.80 (18.24)	–	–	–
		Omega–3 PUFAs 1 μg/mL	47.56 (16.80)	17.32 (14.91)	13.10 (6.15)	77.98 (24.30)	0.17	16.0 (33.9)	0.23
		Omega–3 PUFAs 25 μg/mL	44.28 (13.46)	17.78 (12.47)	12.77 (6.66)	74.83 (21.66)	0.28	10.5 (26.1)	0.48
		Omega–3 PUFAs 50 μg/mL	45.21 (13.86)	17.47 (12.38)	13.05 (7.23)	75.72 (22.09)	0.37	11.9 (25.8)	0.43
	24 h	No omega–3 PUFAs	54.48 (18.86)	30.38 (20.20)	30.03 (17.42)	113.06 (13.73)	–	–	–
		Omega–3 PUFAs 1 μg/mL	58.60 (21.70)	29.76 (21.72)	29.20 (16.45)	117.56 (25.72)	0.60	4.6 (23.0)	0.55
		Omega–3 PUFAs 25 μg/mL	51.28 (21.52)	30.20 (20.70)	30.20 (20.70)	113.01 (20.51)	0.99	−0.3 (13.3)	0.94
		Omega–3 PUFAs 50 μg/mL	54.99 (17.74)	31.11 (21.97)	29.78 (21.37)	115.89 (17.55)	0.33	2.3 (8.4)	0.41

C2, acetate; C3, propionate; C4, butyrate; PUFAs, polyunsaturated fatty acids; SCFAs, short-chain fatty acids.

1 Mean (standard deviation) SCFA level (mmol/L) for *n* = 10 participants.

2 Paired *t*-test comparing total SCFA level or % change with control (no omega-3 PUFAs).

3 Mean (standard deviation) % change of total SCFA level from the control (no omega-3 PUFAs) value.

The presence of omega-3 PUFAs in 0.01 mg/mL inulin-containing fermentations was associated with an omega-3 PUFAs concentration-dependent increase in total SCFAs (Table 4 and Figure 1A), with a respective mean increase of 5.26 (1 μg/mL), 9.79 (25 μg/mL), and 12.89 (50 μg/mL) mmol/L total SCFAs at 24 h, amounting to respective % increases of 8.4 [SD 21.1]%, 15.3 [17.6]%, and 19.3 [20.3]% compared with inulin-only fermentations (Table 4 and Figure 1A), which reached statistical significance (*P* ≤ 0.02) for omega-3 PUFA concentrations equal to and above 25 μg/mL (Table 4).

The largest increase in 0.01 mg/mL inulin-induced SCFA production was observed for butyrate at 24 h in the presence of 50 μg/mL omega-3 PUFAs. The mean difference in butyrate concentration at 24 h between the inulin (no omega-3 PUFAs) control (11.77 [3.08] mmol/L) and the inulin fermentation in the presence of 50 μg/mL omega-3 PUFAs (15.82 [5.30] mmol/L) was 4.05 mmol/L (*P* = 0.02), which equated to a mean 19.3% increase compared with control (*P* = 0.01). A statistically significant increase was also observed in the presence of 25 μg/mL omega-3 PUFAs when compared to the inulin-only fermentation at 24 h (14.83 [4.78] mmol/L; *P* = 0.02). The increase in acetate and propionate levels in inulin-containing fermentations in the presence of omega-3 PUFAs did not reach statistical significance (Table 4).

Although there was a consistent induction of SCFA production by inulin for all donors, inter-individual variability in total SCFA production in 0.01 mg/mL inulin-containing fermentations between individual participants was clearly evident, with the total SCFA level at 24 h ranging from 66.41 mmol/L (P7) to 119.88 mmol/L (P5) in the presence of 50 μg/mL omega-3 PUFAs. The mean % increase in total SCFAs at 24 h in the presence of 50 μg/mL omega-3 PUFAs was 19.3%, ranging from 14.8% to 48.1% in individual participants. The % increase in total SCFA level was statistically significant for 25 μg/mL (*P* = 0.02) and 50 μg/mL omega-3 PUFAs at 24 h (*P* = 0.01; Table 4). Expressed as a proportion of total SCFAs, the presence of 0.01 mg/mL inulin was associated with an increase in propionate and butyrate, at the expense of acetate, particularly at 24 h (Figure 1B and C).

Addition of 0.02 mg/mL inulin to fermentations was associated with increased production of all three major SCFAs compared with the lower concentration of inulin, in the absence and presence of exogenous omega-3 PUFAs (Table 4). This was particularly evident for butyrate, levels of which after 24 h incubation were approximately double those in reactions in the presence of 0.01 mg/mL inulin (Table 4) leading to butyrate being a higher proportion of total SCFAs than other fibre fermentations (Figure 1B and C). Incubation with the higher inulin concentration was associated with higher inter-individual variability in SCFA production than fermentations in the presence of 0.01 mg/mL inulin (Table 4 and Supplementary Figures 3 and 4). In contrast to the interaction between 0.01 mg/mL inulin and omega-3 PUFAs, there was no statistically significant omega-3 PUFAs concentration-dependent increase in SCFA levels when 0.02 mg/mL inulin was present as substrate (Table 4 and Figure 1A). This may reflect near-maximal SCFA production by *in vitro* fermentations in the presence of higher concentration of inulin.

We also measured EPA and DHA levels over time during *in vitro* fermentations (*n* = 5 at each time point). In the absence of exogenous inulin, the mean (SD) EPA and DHA level decreased to 36.7 (21.6)% and 38.2 (29.2)% of baseline levels, respectively, at 8 h, with a further statistically insignificant decrease to 26.5 (20.4)% and 24.0 (23.8)% of baseline levels of EPA and DHA, by 24 h (*P* > 0.05 for comparison with % levels at 8 h, in each case). In inulin (0.01 mg/mL)-containing reactions, the reduction in EPA and DHA concentrations at 8 h was less than in the absence of inulin (53.1 [36.9]% and 50.0 [23.4]% of baseline levels, respectively), but values were similar in inulin-containing reactions compared with no-inulin reactions at the later 24-h time point (23.5 [11.3]% and 19.7 [12.9]% of baseline levels) (*P* = 0.1 and 0.03 for the comparison with % levels at 8).

### The effect of omega-3 PUFAs on SCFA production during in vitro fermentation with other dietary fibres

We next investigated an interaction between omega-3 PUFAs and other dietary fibres (Figure 1A and Supplementary Figure 5). In comparison to inulin, wheat bran was fermented to a lesser degree within the *in vitro* fermentation model, with no major change in the proportions of the major individual SCFAs (Figure 1A–C and Supplementary Tables 1 and 2). The mean pH of fermentations containing 0.01 mg/mL wheat bran was 7.46 (SD 0.06) at baseline, which decreased to 6.38 (SD 0.26) at 24 h. There was no statistically significant change in either pH or in total or individual SCFA levels in fermentations at 8 or 24 h in the presence of omega-3 PUFAs (Supplementary Tables 1 and 2). The presence of omega-3 PUFAs did not alter the proportion of individual SCFAs in wheat bran fermentations, which was predominantly acetate (Figure 1B and C).

The addition of pectin (0.01 mg/mL) to the *in vitro* fermentation model was associated with a mean decrease in pH from 7.36 (0.09) at baseline to 5.44 (0.21) at 24 h (Supplementary Table 3). Overall, pectin induced SCFA production to a similar level to the lower concentration of inulin and more than wheat bran (Figure 1A). In the presence of pectin substrate, the greatest acidification and highest SCFA production were associated with 1 μg/mL omega-3 PUFAs rather than higher omega-3 PUFA concentrations (Supplementary Tables 3 and 4). The presence of omega-3 PUFAs (1 μg/mL) in pectin-containing fermentations was associated with the largest decrease in pH at 24 h (5.40 [0.19]) compared to pectin alone (5.44 [0.18]), a difference that just missed statistical significance (*P* = 0.07; Supplementary Table 3). Maximal acidification in the presence of 1 μg/mL omega-3 PUFAs was reflected in an increase in total SCFAs level at 24 h (88.17 [11.77] mmol/L) compared with the pectin-alone fermentation (83.37 [12.01] mmol/L; mean 6.4% increase, *P* = 0.004), unlike higher omega-3 PUFA concentrations (Supplementary Table 4). The presence of omega-3 PUFAs did not alter the proportion of individual SCFAs in pectin fermentations, which was predominantly acetate (Figure 1B and C).

### *Changes to the faecal microbiome associated with inulin and omega-3 PUFAs in the* in vitro *fermentation model*

Given that an omega-3 PUFA concentration-dependent increase in SCFA production was restricted to fermentations using 0.01 mg/mL inulin, we focused the microbiome analysis on the model that examined the interaction of omega-3 PUFAs with 0.01 mg/mL inulin. This included baseline and 24-h control (no omega-3 PUFA or fibre) fermentations, as well as fermentations in the presence of 0.01 mg/mL inulin, alone and in combination with 50 μg/mL omega-3 PUFAs. Six participants (P1, P2, P5, P6, P8, and P9: 2 male, 4 female; 5 White British, and 1 Arab ethnicity) were selected based on the highest increase in total SCFAs in their control fermentation (Supplementary Figure 2).

**Figure 2.**
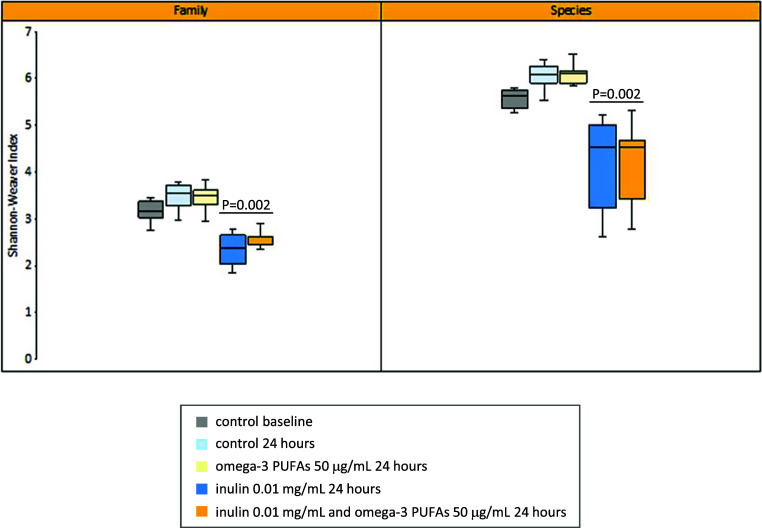
The Shannon–Weaver diversity index of *in vitro* fermentations at the Family and Species taxonomic level. *Note*: Summary data for fermentations in the absence or presence of 0.01 mg/mL inulin and 50 μg/mL omega-3 PUFAs are presented for six participants. The box-plot line denotes the median value for six participants with boxes extending between the 25th and 75th percentile values. The minimum and maximum data points are denoted by bars. *P* = 0.002 for the comparison of fermentations with and without exogenous inulin at both Family and Species level, in both the absence or presence of omega-3 PUFAs (Mann–Whitney *U* test).

The Shannon–Weaver diversity index at the Family and Species taxonomic level increased over time in the control (no omega-3 PUFA or fibre) 24-h fermentation model, but the difference from baseline did not reach statistical significance (median 3.17 compared with 3.54 [*P* = 0.24] at the Family level; median 5.58 compared with 6.03 [*P* = 0.18] at the Species level; Figure 2). The presence of 50 μg/mL omega-3 PUFAs did not alter microbiome diversity in the *in vitro* model in the absence of exogenous inulin (median Shannon–Weaver index 3.49 [Family-level] and 6.05 [Species-level]; Figure 2). Exposure to inulin was associated with a significant reduction in diversity independently of the presence or absence of exogenous omega-3 PUFAs (median Shannon–Weaver index in the absence of exogenous omega-3 PUFAs 2.37 [Family-level] and 4.51 [Species-level]; *P* = 0.002 for both comparisons with control [no inulin] reactions at 24 h; Figure 2).

**Figure 3.**
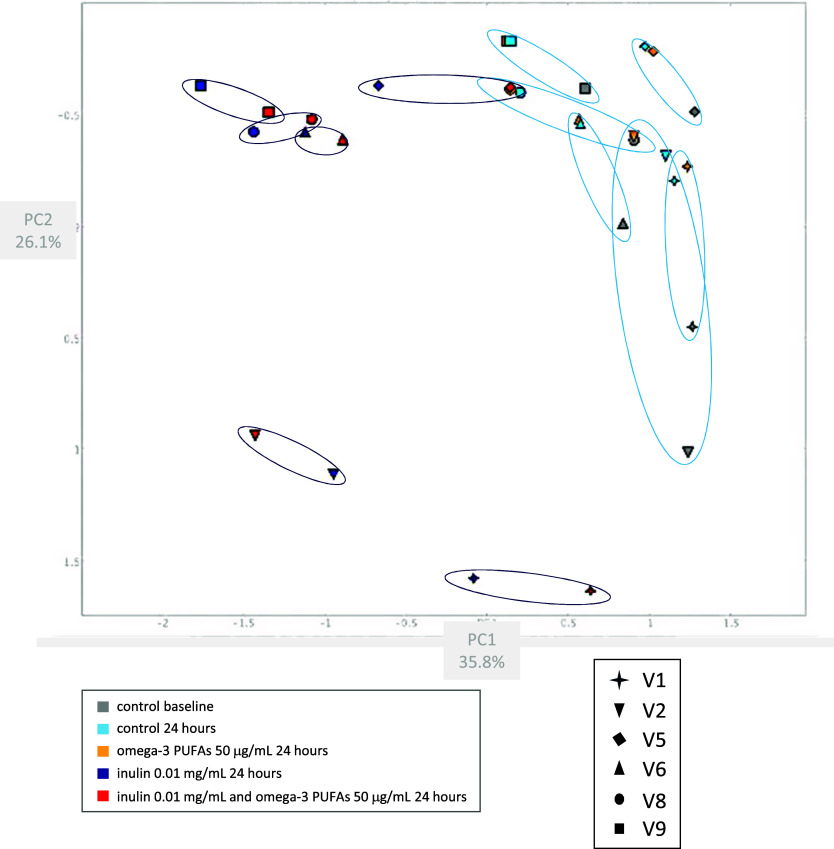
Bray–Curtis Principal Coordinate Analysis of the microbiome composition of *in vitro* fermentation reactions. *Note*: Faecal samples from six participants (P; each denoted by a different shaped symbol) were analysed. The baseline fermentation profile for each participant (grey) is grouped with equivalent data from the fermentation reaction after 24 h in the absence (light blue) or presence (amber) of 50 μg/mL omega-3 PUFAs by a light blue ellipse. Fermentation reactions from the same participants after 24-h incubation with 0.01 mg/mL inulin, in the absence (dark blue) or presence (red) of 50 μg/mL omega-3 PUFAs are grouped by a dark blue ellipse.

Bray–Curtis PCoA mirrored the diversity data with stability of the microbiome composition in the *in vitro* fermentation model over 24 h (Figure 3), and minimal change in composition in the presence of omega-3 PUFAs alone for 24 h (Figure 3). By contrast, there was a marked change in microbiome composition when 0.01 mg/mL inulin was added for 24 h compared with the paired control (no omega-3 PUFA or fibre) fermentation (Figure 3). There was little shift in the composition of the inulin fermentations when omega-3 PUFAs were also present (Figure 3).

**Figure 4.**
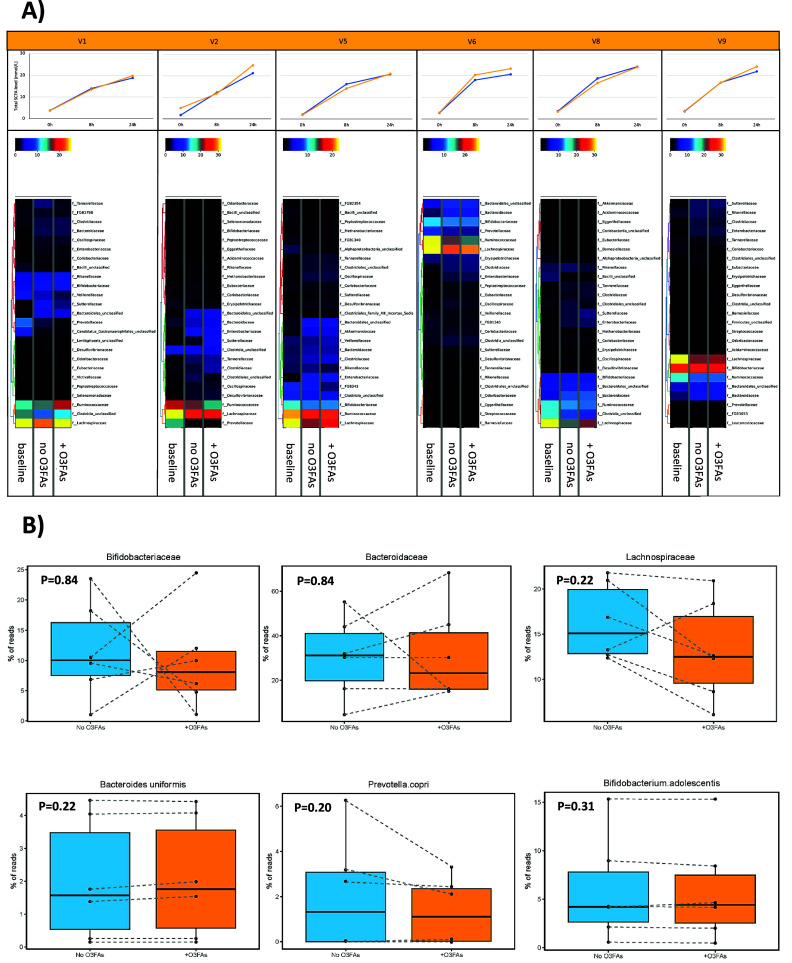
The effect of omega-3 PUFAs on the faecal microbiota during *in vitro* fermentation for 24 h. *Note*: (A) Heatmaps of relative taxonomic abundance for the top 25 most abundant bacterial Families in *in vitro* fermentations of faecal samples from six participants (labelled V1, V2, V5, V6, V8, and V9). For each participant, abundance is compared between the fermentation reaction at baseline and the fermentation at 24 h in the absence (no O3FAs) and presence of 50 μg/mL omega-3 PUFAs (+O3FAs). For each participant, abundance is normalised to the lowest read count in the baseline sample. Each heatmap is accompanied by the respective plot of total SCFAs level in the fermentation reaction over time (blue, no omega-3 PUFAs; yellow, 50 μg/mL omega-3 PUFAs). (B) Percentage read counts of representative bacterial families and species in fermentation reactions after 24-h incubation in the absence (light blue box) or presence (yellow box) of 50 μg/mL omega-3 PUFAs (O3FAs). For each boxplot, the line denotes the median value, with the box extending between the 25th and 75th percentile values. The minimum and maximum data points are denoted by bars, with any outlying value marked separately. Data points from each participant are joined by a dashed line. Pairwise comparisons of bacterial abundance in the absence or presence of omega-3 PUFAs were performed using the Wilcoxon signed-rank test.

**Figure 5.**
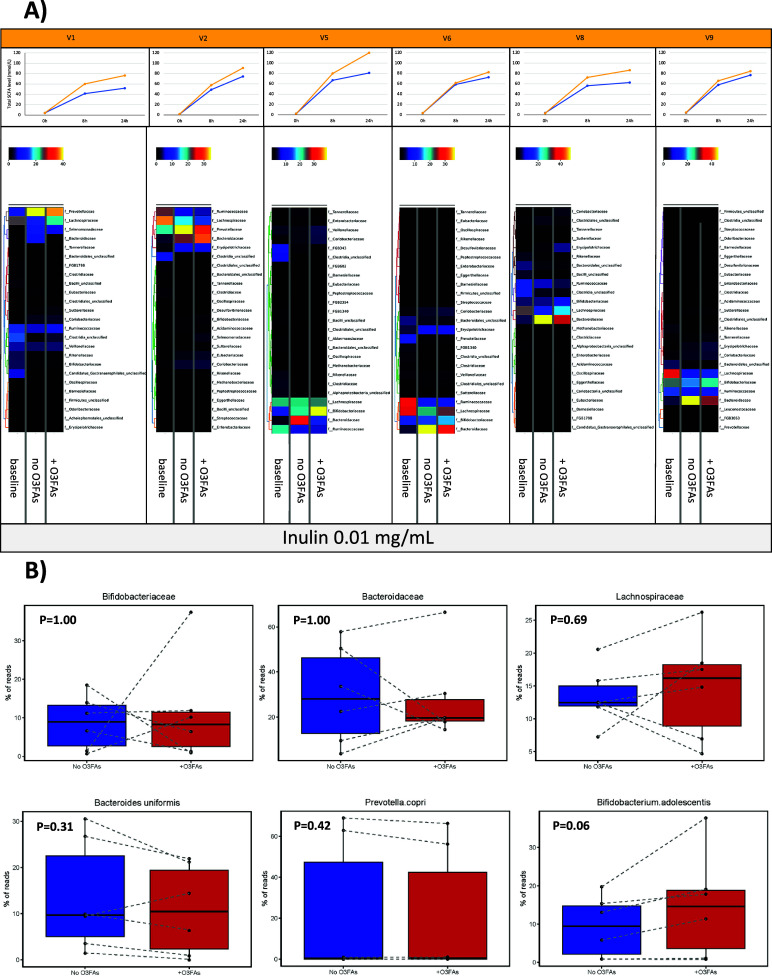
The effect of omega-3 PUFAs on the faecal microbiota during *in vitro* fermentation in the presence of 0.01 mg/mL inulin for 24 h. *Note*: (A) Heatmaps of relative taxonomic abundance for the top 25 most abundant bacterial Families in *in vitro* fermentations of faecal samples from six participants (labelled V1, V2, V5, V6, V8, and V9). For each participant, abundance is compared between the fermentation reaction at baseline and the fermentation with 0.01 mg/mL inulin at 24 h in the absence (no O3FAs) and presence of 50 μg/mL omega-3 PUFAs (+O3FAs). For each participant, abundance is normalised to the lowest read count in the baseline sample. Each heatmap is accompanied by the respective plot of total SCFAs level in the fermentation reaction over time (blue, no omega-3 PUFAs; yellow, 50 μg/mL omega-3 PUFAs). (B) Percentage read counts of representative bacterial families and species in fermentation reactions after 24-h incubation in the absence (dark blue box) or presence (red box) of 50 μg/mL omega-3 PUFAs (O3FAs). For each boxplot, the line denotes the median value, with the box extending between the 25th and 75th percentile values. The minimum and maximum data points are denoted by bars, with any outlying value marked separately. Data points from each participant are joined by a dashed line. Pairwise comparisons of bacterial abundance in the absence or presence of omega-3 PUFAs were performed using the Wilcoxon signed-rank test.

The relative difference in microbiota profile at the Family taxonomic level between samples from each participant is summarised in Figures 4 and 5. In control (no omega-3 PUFAs) fermentations, pairwise comparison showed that there was a decrease in abundance of *Lachnospiraceae* and *Ruminococcaceae* at 24 h compared with the baseline profile, which was evident for all participants (Figure 4A). A decrease in *Prevotellaceae* during *in vitro* fermentation was also observed in participants who had *Prevotellaceae* evident at baseline (P1 and P2; Figure 4A). There were no marked changes in the microbiota profile at either Family or Species level in the presence of omega-3 PUFAs compared with the paired control (no omega-3 PUFAs) fermentation (Figure 4A and B). In several participants, there was abrogation of the reduction in *Lachnospiraceae* and *Ruminococcaceae* abundance over time in fermentations in the presence of 50 μg/mL omega-3 PUFAs compared with that seen in the control fermentation (see P1, P5, and P8; Figure 4A). There was no statistically significant difference in the % abundance of *Bacteroidaceae* or *Bifidobacteriaceae*, or representative Species from these taxa, in fermentations completed in the presence or absence of omega-3 PUFAs for each participant (Figure 4B). There was also no consistent relationship between the change in abundance of any of the major bacterial Families and the increase in SCFA levels during *in vitro* fermentations in the absence of an exogenous dietary fibre (Figure 4A and B).

During *in vitro* fermentation for 24 h in the presence of 0.01 mg/mL inulin, there was also a consistent decline in *Lachnospiraceae* and *Ruminococcaceae* abundance when paired samples from the same participant were compared (Figure 5A). However, in contrast to fermentations carried out in the absence of inulin fibre in the same participants, there was an increase in *Bacteroidaceae* in all participants during fermentation for 24 h in the presence of inulin (Figure 5A). The two participants, who demonstrated a decrease in *Prevotellaceae* abundance during fermentation in the absence of inulin, actually demonstrated a relative increase in *Prevotellaceae* in the *in vitro* fermentations to which inulin had been added. (Figure 5A). The presence of 50 μg/mL omega-3 PUFAs in inulin-containing fermentations was associated with a smaller increase in *Bacteroidaceae* compared with control (no omega-3 PUFAs) fermentations (Figure 5A). There was also an increase in *Bifidobacteriaceae* in inulin-containing reactions that also included omega-3 PUFAs compared with the control fermentation (Figure 5A). In the presence of inulin, addition of omega-3 PUFAs was associated with a smaller increase in *Prevotellaceae* than fermentations in the absence of omega-3 PUFAs (Figure 5A), but any difference did not reach statistical significance due to the small number of fermentations with measurable *Prevotella* species (Figure 5B). Abundance data expressed as the % read count confirmed inter-individual variability in % abundance at Family and Species level in the *in vitro* fermentation model (Figure 5B). The increase in *Bifidobacterium adolescentis* abundance in omega-3 PUFA-containing fermentations compared with paired control fermentations just failed to reach pre-specified statistical significance for the four participants with detectable *B. adolescentis* (*P* = 0.06; Figure 5B). No relationship was evident between the change in abundance of any of the bacterial Families and the increase in SCFA levels during *in vitro* fermentation in the presence of 0.01 mg/mL inulin (Figure 5A and B).

Functional analysis of changes in expression of SCFA-metabolism genes related to exposure to omega-3 PUFAs (50 μg/mL) and/or inulin (0.01 mg/mL) did not show any marked difference in abundance of selected SCFA-metabolising genes according to the presence of either omega-3 PUFAs or inulin alone for 24 h (Supplementary Figure 6). However, in inulin-containing reactions, there was a suggestion that the presence of omega-3 PUFAs was associated with increased abundance of *phosphate acetyltransferase*, *acetate kinase*, and *short-chain acyl-CoA dehydrogenase* genes (Supplementary Figure 6).

## Discussion

We report that mixed omega-3 PUFAs, at concentrations found in the distal small intestine after short-term oral dosing (Nana et al., 2021), are associated with a small (10–20%) increase in SCFA levels in a static *in vitro* fermentation model of the human colon. Augmentation of SCFA levels attained in the *in vitro* model by omega-3 PUFAs was evident for fermentations in the presence of 0.01 mg/mL inulin and also pectin (for low concentration omega-3 PUFAs). The presence of omega-3 PUFAs was not associated with any marked or consistent change in the faecal microbiota profile during fermentation in the absence of exogenous inulin for 24 h.

We used an established static, anaerobic *in vitro* fermentation model developed by the Edwards laboratory (Harris et al., 2017; Havlik et al., 2020), which has been used repeatedly to investigate the effect of nutrients on SCFA production (Harris et al., 2019; Mansoorian et al., 2019; Mhd Jalil AM et al., 2019). This high-throughput, small-volume model allowed us to test two interventions (omega-3 PUFAs and a dietary fibre) in multiple simultaneous reactions using a single faecal sample from each participant. However, the static model is limited by substrate depletion and the accumulation of microbial metabolites that would not occur *in vivo*, and the model most closely reflects the proximal colon, as opposed to the more distal large intestine, where dietary fibres may be metabolised differently (Isenring et al., 2023). Significant inter-individual variability in the baseline taxonomic profile was evident, in combination with a major shift in profile associated with the presence of inulin in fermentations from all participants. Although the overall microbiota profile was stable over 24 h, as shown by the small shift between paired baseline and 24-h samples by Principal Component Analysis, a consistent feature inherent to the *in vitro* fermentation model was the reduction in abundance of butyrate-producing *Lachnospiraceae* and *Ruminococcaceae* families over 24 h for each participant, which was independent of exogenous addition of either inulin or omega-3 PUFAs. However, an increase in *Bifidobacteriaceae* abundance (and of *Prevotellaceae* in participants that had prevalent *Prevotellaceae* at baseline) in inulin-containing reactions suggests that the *in vitro* fermentation model mirrors at least some of the microbiome changes induced by inulin *in vivo* (Baxter et al., 2019; Hoffman et al., 2019).

It is important to emphasise that the changes in microbiota profile associated with the addition of omega-3 PUFAs and/or inulin to *in vitro* fermentations were only evident for comparisons of samples for each participant, given the larger inter-individual variability in microbiota composition between participants. Changes in SCFA levels and the microbiota profile associated with the presence of inulin, including an increase in abundance of *Bacteroidaceae*, were quantitatively higher than any changes associated with the addition of mixed omega-3 PUFAs to fermentation reactions, consistent with the powerful prebiotic effect of this fibre (Le Bastard et al., 2020; Riva et al., 2023).

The modest but statistically significant increase in SCFA levels that was observed in the absence of omega-3 PUFAs is consistent with previous studies using the same static *in vitro* fermentation model and similar concentrations of dietary fibres (Havlik et al., 2020). The small increase in SCFA levels observed in the presence of omega-3 PUFAs above values in reactions in the absence of exogenous omega-3 PUFAs at 24 h is also consistent with limited data from other studies using human faecal samples (Hull & Sun, 2025; Salsinha et al., 2025). Rehman et al. (2024) examined the effects of EPA and DHA on a single faecal inoculum from one healthy participant in a static anaerobic model for 48 h. High (mg/mL) concentrations of EPA and DHA were tested separately and were associated with a 20–25% increase in total SCFAs concentration at 24 and 48 h (Rehman et al., 2024). This model showed minimal change in major SCFA-producing taxa, including *Lactobacillus*, *Bifidobacterium*, and *Akkermansia* (Rehman et al., 2024). Omega-3 PUFAs have also been added to a dynamic, chemostat human gut microbiota model (M-SHIME®), testing the microbiota in replicate samples from a single healthy male donor (Roussel et al., 2022). After a 14-day stabilisation period, a mixed omega-3 PUFA intervention (990 mg EPA and 990 mg DHA triglycerides daily into the SHIME® stomach) was administered for 7 days, which was associated with increased SCFA levels in ileal and colonic vessels (Roussel et al., 2022). An increase in the propionate/butyrate ratio was noted, which was significantly correlated with an increase in *Verrucomicrobiae* and *Desulfovibrionia* classes (Roussel et al., 2022). An increase in faecal SCFA levels after oral administration of mixed omega-3 PUFAs has also been reported in several rodent studies (Caesar et al., 2015; Liu et al., 2020; Tao et al., 2021; Cao et al., 2025).

In keeping with the small increase in SCFA levels associated with the presence of omega-3 PUFAs in our *in vitro* fermentation model, functional KEGG analysis of the metagenomic data suggested that there was no marked increase in expression of SCFA-metabolising enzymes. It is possible that a contribution to higher SCFA levels in omega-3 PUFA-containing fermentations is the beta-oxidation of exogenous omega-3 PUFA substrate. Alternatively, omega-3 PUFAs could increase expression of bacterial inulinases that are necessary for the release of fructose monomers from inulin or increase the inulin binding capacity of bacteria (Singh et al., 2020; Riva et al., 2023).

Direct comparison of SCFA levels measured in *in vitro* fermentation models with clinical data is challenging, given that faecal and circulating blood SCFA levels are not representative of SCFA production, allowing for systemic absorption and rapid metabolic utilisation of SCFAs (Vogt & Wolever, 2003). One study measured plasma SCFAs in healthy human individuals taking either mixed omega-3 PUFAs (500 mg/day, including 165 mg EPA and 110 mg DHA) or inulin (20 g/day) for 6 weeks (Vijay et al., 2021). Although total SCFA levels were not reported, there was a statistically significant increase in plasma isobutyrate and isovalerate levels (and a near-significant increase in plasma butyrate levels) following the omega-3 PUFA intervention, which mirrored findings in the inulin intervention group (Vijay et al., 2021).

The modest microbiota changes that were associated with the addition of physiologically relevant concentrations of omega-3 PUFAs to *in vitro* fermentations mirror the faecal microbiome findings from clinical intervention studies of oral omega-3 PUFAs (Watson et al., 2018; Vijay et al., 2021; Huang et al., 2024; Lu et al., 2024). These clinical studies have consistently shown little or no change in alpha- or beta-diversity in faecal samples, although differences in individual bacterial taxa abundance have been reported in a study-specific manner (Watson et al., 2018; Vijay et al., 2021; Huang et al., 2024; Lu et al., 2024). Abrogation of the decrease in SCFA-producing *Ruminococcaceae* and *Lachnospiraceae* abundance *in vitro* in the presence of 50 μg/mL omega-3 PUFAs is consistent with a study of middle-aged and elderly female twins, in whom plasma DHA levels were positively correlated with abundance of *Lachnospiraceae* in faecal samples (Menni et al., 2017).

We also provide novel comparative data regarding other soluble (pectin) and insoluble (wheat bran) fibres. A small interaction between pectin and low concentration omega-3 PUFAs, but not wheat bran, in the *in vitro* fermentation model highlights that any interaction between omega-3 PUFAs and fibre intake *in vivo* is fibre-specific (Baxter et al., 2019). Our data are consistent with other *in vitro* models that have provided a direct comparison of SCFA production between inulin and other dietary fibres (Poeker et al., 2018).

Strengths of our study include the use of concentrations of omega-3 PUFAs and dietary fibres that are relevant to the bioavailability of these nutrients in the proximal colon after purified omega-3 PUFA supplementation and realistic dietary fibre ingestion (Nana et al., 2021; Thomson et al., 2021) in an established *in vitro* model (Harris et al., 2017; Havlik et al., 2020).

A limitation of the *in vitro* model is that we did not supplement the *in vitro* reactions with omega-3 PUFAs during the 24-h fermentation period after the addition of an omega-3 PUFA bolus at baseline. Therefore, omega-3 PUFA concentrations decreased by ~75–80% during the 24-h experiment, which might relate to microbial metabolism of long-chain PUFAs. Fluctuating omega-3 PUFA levels over time in the intestinal tract *in vivo* may reflect differential exposure over time related to oral omega-3 PUFA intake, as observed in our ileostomy study (Nana et al., 2021). Importantly, there was no difference in apparent degradation of omega-3 PUFAs in the absence or presence of exogenous inulin. Another limitation shared by other static *in vitro* fermentation models is that acidification of the reaction content over time (especially in the presence of exogenous fibres) could alter microbial content and metabolism.

Our study adds to the evidence that oral omega-3 PUFA intake will be associated with increased colonic SCFA levels, especially in the presence of inulin. This *in vitro* evidence should prompt a proof-of-concept clinical study that formally studies the effect of a combined omega-3 PUFA and inulin intervention on the gut microbiome and SCFA production *in vivo.* Formal testing of an omega-3 PUFA-fibre combination prebiotic supplement (or an equivalent dietary intervention) will be required to determine the efficacy and safety of a strategy to drive SCFA production for gut health and improved disease outcomes (Kaźmierczak-Siedlecka et al., 2022). A recent mouse study reported that a high (20%) inulin-containing diet, which increased faecal butyrate levels, was associated with higher tumour number, in *Apc*^Min/+^ and azoxymethane-induced mouse models of intestinal tumorigenesis (Yang et al., 2024), a finding consistent with the notion that, in some circumstances, butyrate may drive tumorigenesis rather than have dominant anti-cancer properties (Bultman & Jobin, 2014).

## Supporting information

10.1017/gmb.2025.10016.sm001 Aldoori et al. supplementary material Aldoori et al. supplementary material

## Supplementary material

The supplementary material for this article can be found at http://doi.org/10.1017/gmb.2025.10016.
