# Supplementary material for: The effect of omega-3 polyunsaturated fatty acids on short-chain fatty acid production and the gut microbiome in an in vitro colonic fermentation model
Source: Gut Microbiome (Camb). 2026 Jan 6;7:e1. doi: 10.1017/gmb.2025.10016 (PMC12835959; doi:10.1017/gmb.2025.10016)
Supplement: Aldoori et al. supplementary material [file S2632289725100169sup001.zip › O3FAs in vitro model paper supplementary figure 1.pptx]

## Slide 1
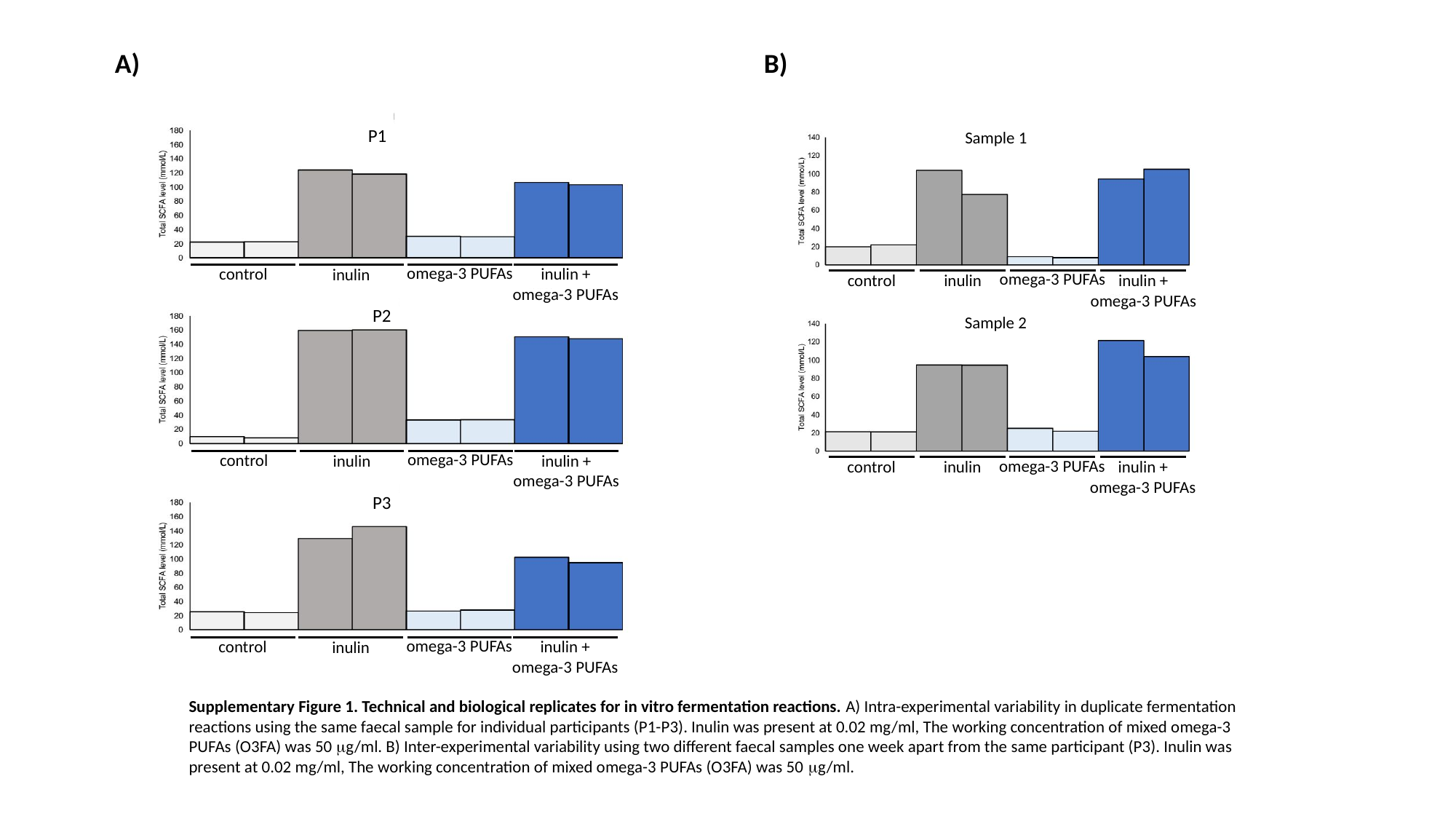

A)
B)
Sample 1
Sample 2
P1
omega-3 PUFAs
control
inulin +
omega-3 PUFAs
inulin
omega-3 PUFAs
control
inulin +
omega-3 PUFAs
inulin
P2
omega-3 PUFAs
control
inulin +
omega-3 PUFAs
inulin
omega-3 PUFAs
control
inulin +
omega-3 PUFAs
inulin
P3
omega-3 PUFAs
control
inulin +
omega-3 PUFAs
inulin
Supplementary Figure 1. Technical and biological replicates for in vitro fermentation reactions. A) Intra-experimental variability in duplicate fermentation reactions using the same faecal sample for individual participants (P1-P3). Inulin was present at 0.02 mg/ml, The working concentration of mixed omega-3 PUFAs (O3FA) was 50 mg/ml. B) Inter-experimental variability using two different faecal samples one week apart from the same participant (P3). Inulin was present at 0.02 mg/ml, The working concentration of mixed omega-3 PUFAs (O3FA) was 50 mg/ml.
