# Supplementary material for: The effect of omega-3 polyunsaturated fatty acids on short-chain fatty acid production and the gut microbiome in an in vitro colonic fermentation model
Source: Gut Microbiome (Camb). 2026 Jan 6;7:e1. doi: 10.1017/gmb.2025.10016 (PMC12835959; doi:10.1017/gmb.2025.10016)
Supplement: Aldoori et al. supplementary material [file S2632289725100169sup001.zip › O3FAs in vitro model paper supplementary figure 2.pptx]

## Slide 1
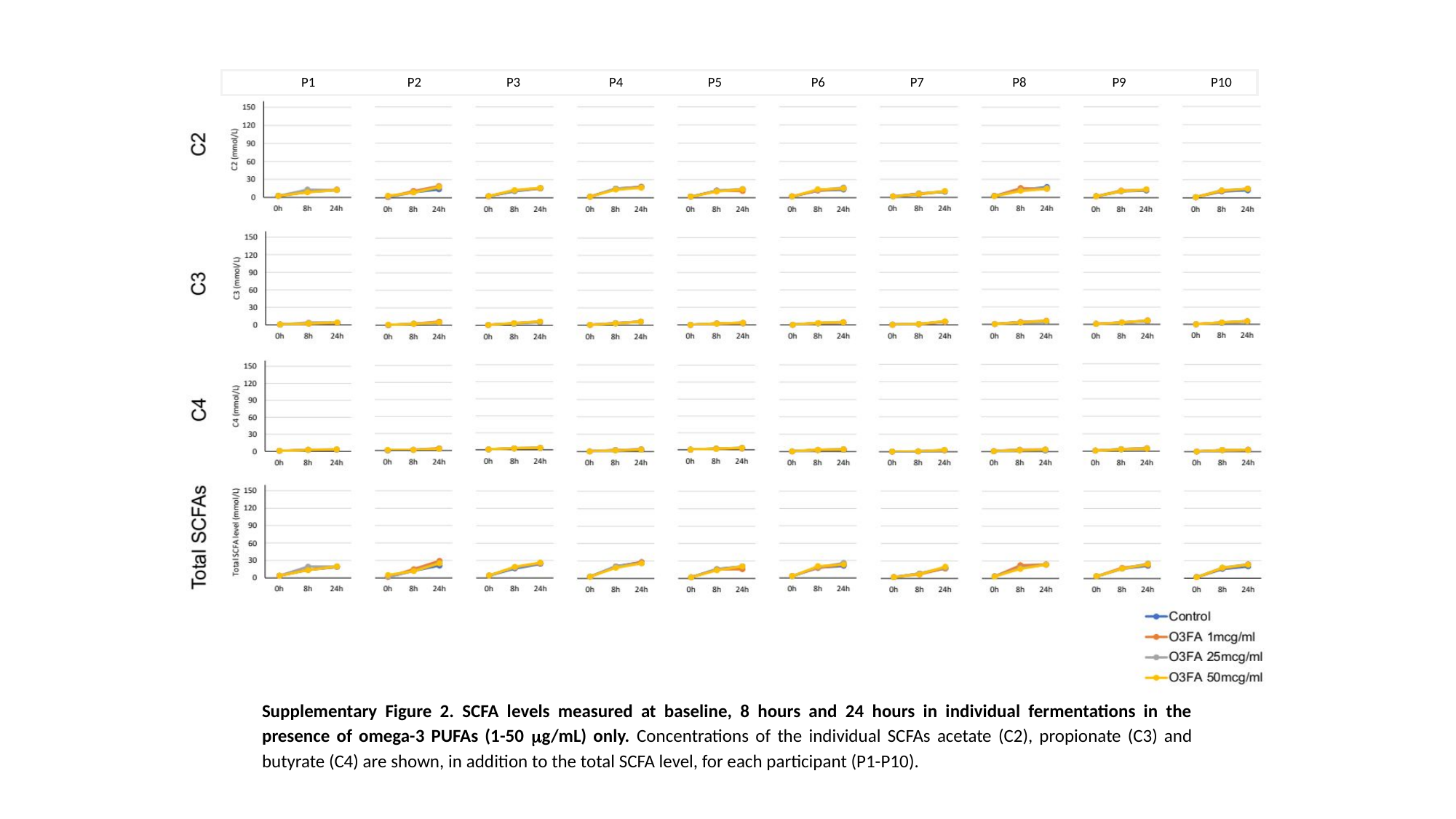

P1
P2
P3
P4
P5
P6
P7
P8
P9
P10
Supplementary Figure 2. SCFA levels measured at baseline, 8 hours and 24 hours in individual fermentations in the presence of omega-3 PUFAs (1-50 mg/mL) only. Concentrations of the individual SCFAs acetate (C2), propionate (C3) and butyrate (C4) are shown, in addition to the total SCFA level, for each participant (P1-P10).
