# Supplementary material for: The effect of omega-3 polyunsaturated fatty acids on short-chain fatty acid production and the gut microbiome in an in vitro colonic fermentation model
Source: Gut Microbiome (Camb). 2026 Jan 6;7:e1. doi: 10.1017/gmb.2025.10016 (PMC12835959; doi:10.1017/gmb.2025.10016)
Supplement: Aldoori et al. supplementary material [file S2632289725100169sup001.zip › O3FAs in vitro model paper supplementary figure 5.pptx]

## Slide 1
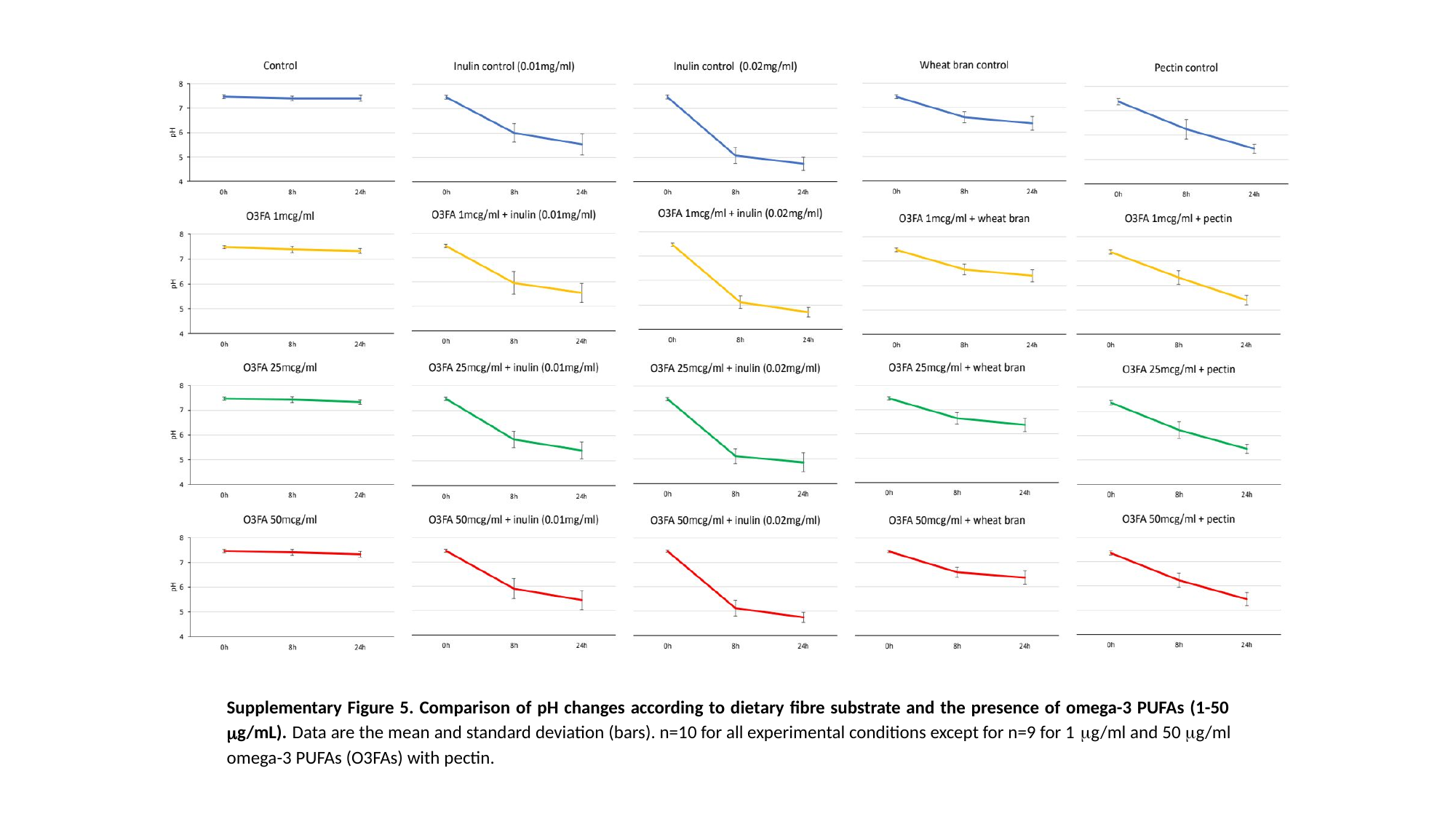

Supplementary Figure 5. Comparison of pH changes according to dietary fibre substrate and the presence of omega-3 PUFAs (1-50 mg/mL). Data are the mean and standard deviation (bars). n=10 for all experimental conditions except for n=9 for 1 mg/ml and 50 mg/ml omega-3 PUFAs (O3FAs) with pectin.
