# Supplementary material for: The effect of omega-3 polyunsaturated fatty acids on short-chain fatty acid production and the gut microbiome in an in vitro colonic fermentation model
Source: Gut Microbiome (Camb). 2026 Jan 6;7:e1. doi: 10.1017/gmb.2025.10016 (PMC12835959; doi:10.1017/gmb.2025.10016)
Supplement: Aldoori et al. supplementary material [file S2632289725100169sup001.zip › O3FAs in vitro model paper supplementary figure 6.pptx]

## Slide 1
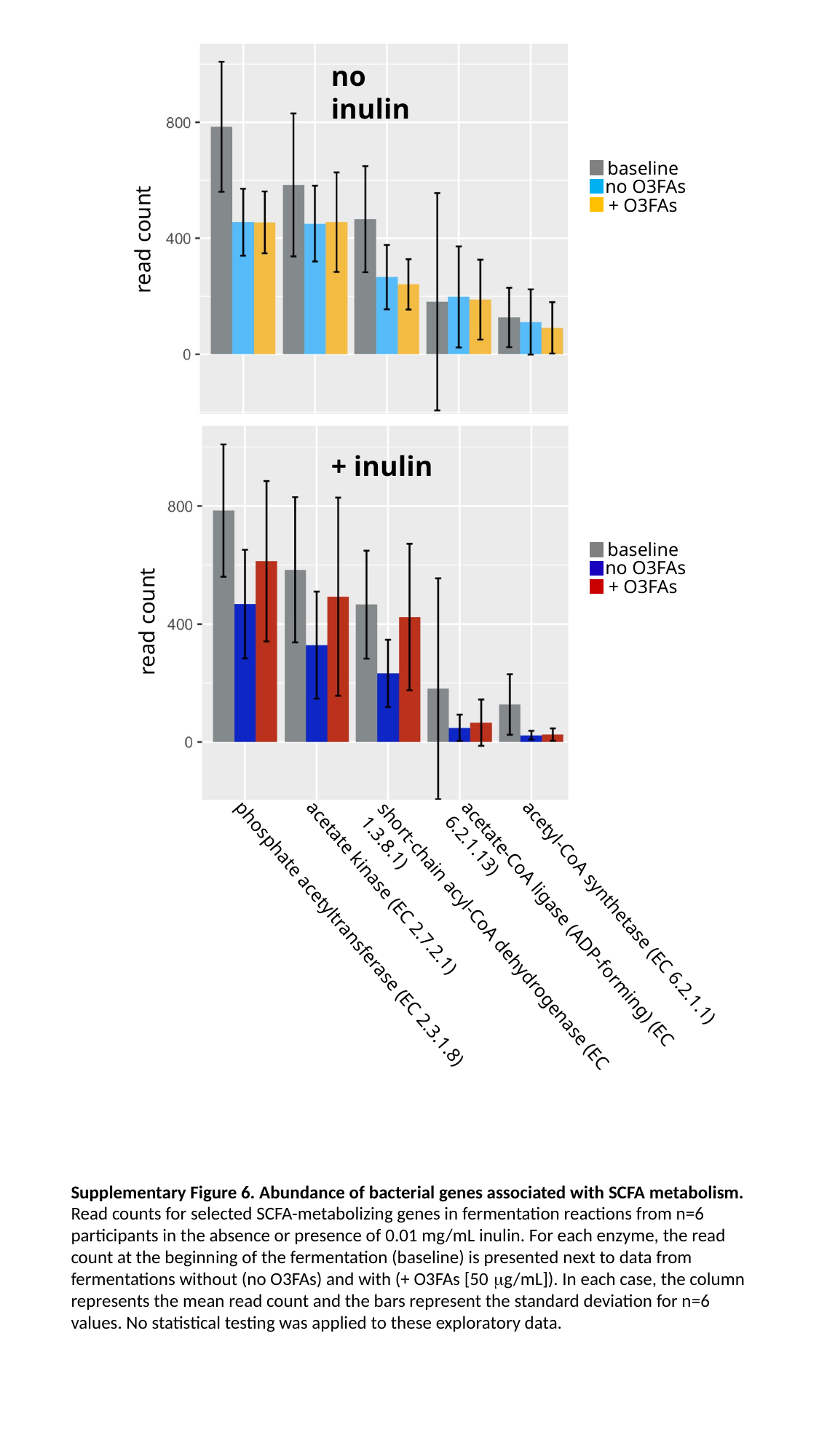

no inulin
read count
baseline
no O3FAs
+ O3FAs
+ inulin
read count
baseline
no O3FAs
+ O3FAs
phosphate acetyltransferase (EC 2.3.1.8)
acetate kinase (EC 2.7.2.1)
acetate-CoA ligase (ADP-forming) (EC 6.2.1.13)
acetyl-CoA synthetase (EC 6.2.1.1)
short-chain acyl-CoA dehydrogenase (EC 1.3.8.1)
Supplementary Figure 6. Abundance of bacterial genes associated with SCFA metabolism. Read counts for selected SCFA-metabolizing genes in fermentation reactions from n=6 participants in the absence or presence of 0.01 mg/mL inulin. For each enzyme, the read count at the beginning of the fermentation (baseline) is presented next to data from fermentations without (no O3FAs) and with (+ O3FAs [50 mg/mL]). In each case, the column represents the mean read count and the bars represent the standard deviation for n=6 values. No statistical testing was applied to these exploratory data.
