# Supplementary material for: The effect of omega-3 polyunsaturated fatty acids on short-chain fatty acid production and the gut microbiome in an in vitro colonic fermentation model
Source: Gut Microbiome (Camb). 2026 Jan 6;7:e1. doi: 10.1017/gmb.2025.10016 (PMC12835959; doi:10.1017/gmb.2025.10016)
Supplement: Aldoori et al. supplementary material [file S2632289725100169sup001.zip › O3FAs in vitro model paper supplementary methods.docx]

**The effect of omega-3 polyunsaturated fatty acids on short-chain fatty acid production and the gut microbiome in an *in vitro* colonic fermentation model. Aldoori *et al***

**Supplementary Methods**

Gas-chromatography-flame ionization detection for quantification of short-chain fatty acids

Authentic short-chain fatty acid (SCFA) standards (see main text) and fermentation fluid samples (800 μL) were acidified with orthophosphoric acid (100 μL) and extracted into the organic phase four times with diethyl-ether. 2-ethyl butyric acid (73.8 mmol/L) in 2 mol/L NaOH was added to all samples as an internal extraction standard.

Measurement of SCFAs was performed using an Agilent 7820A gas chromatography system with an Agilent DB-WAX column (inner diameter 0.53 mm; length 15 m, film thickness 1 μm). The carrier gas (nitrogen) was set at a flow rate of 40 mL/min. Maximum oven temperature was 230^o^C. Analytes were detected by a flame ionisation detector (FID) at a base temperature of 250^o^C, with a hydrogen flow rate of 30 mL/min and an air flow rate of 400 mL/min.

The absolute concentration of each SCFA was calculated from the area under the peak for each SCFA compared with the concentration range of its authentic standard after correction for the extraction efficiency for each sample.

Liquid chromatography-tandem mass spectrometry for measurement of omega-3 polyunsaturated fatty acids

Detailed description of the methods used to extract and quantify long-chain fatty acids is available in reference #1. A nine-member panel of saturated (C16:0 palmitic acid, C18:0 stearic acid), mono-unsaturated (C18:1 oleic acid) and polyunsaturated long-chain fatty acids (PUFAs; C18:2*n*-6 linoleic acid, C20:4*n*-6 arachidonic acid, C18:3*n*-3 alpha-linolenic acid, C20:5*n*-3 eicosapentaenoic acid [EPA], C22:5*n*-3 docosapentaenoic acid, C22:6*n*-3 docosahexaenoic acid [DHA]) were quantified using authentic long-chain fatty acids from Merck Life Sciences.

Total fatty acids (free and esterified) were extracted from fermentation fluid by isopropanol/chloroform extraction, with deuterated alpha-linolenic acid-d_14_ (Cayman Chemical Co.) as an internal standard.  Sample supernatants were evaporated to dryness, reconstituted in acetonitrile and then subjected to acidic saponification with 5 mol/L hydrochloric acid at 80^o^C. Following neutralisation with sodium hydroxide and a second evaporation step, extracted fatty acids were derivatised using 4-[2-(*N*,*N*-dimethylamino)ethylaminosulfonyl]-7-(2-aminoethylamino)-2,1,3-benzoxadiazole (Merck Life Sciences). Analysis was performed using a Waters Alliance 2695 High Pressure LC system in combination with a Waters Micromass Quattro Ultima triple quadrupole mass spectrometer operated in electrospray ionisation positive MRM mode.  Analyte separation was achieved by a gradient LC method using a HiChrom RPB column (2.1 mm x 250 mm, 5 µm) and mobile phase (MP) A (90% water, 10% methanol, 0.1% formic acid) and MPB (90% methanol, 10% water and 0.1% formic acid). Gradient conditions were as follows: Starting at 80% MPB changing to 83% MPB over 8 min, then increasing to 95% MPB at 15 min, remaining at 95% MPB until 17 min before returning to starting conditions at 18 min. The overall run time was 25 min. The flow rate was set at 0.5 ml/min and split post-column with 0.3 ml/min delivered to the MS. Samples were analysed in MRM mode. Instrument settings were capillary voltage, 3 kV; cone energy, 15 eV; collision energy, 25 eV; source temperature, 120^o^C and desolvation temperature, 300^o^C.

EPA and DHA levels were calculated as the percentage respective omega-3 PUFA chromatographic peak area relative to the sum of all the fatty acid peak areas.

**References**

1. Volpato, M., Spencer, J.A., Race, A.D., Munnarini, A., Belluzzi, A., Cockbain, A.J., Hull, M.A, & Loadman, P.M. (2017) A liquid chromatography-tandem mass spectrometry method to measure fatty acids in biological samples. *J Chromatography B*, *1055-1056*, 125-134.
