# Supplementary material for: The effect of omega-3 polyunsaturated fatty acids on short-chain fatty acid production and the gut microbiome in an in vitro colonic fermentation model
Source: Gut Microbiome (Camb). 2026 Jan 6;7:e1. doi: 10.1017/gmb.2025.10016 (PMC12835959; doi:10.1017/gmb.2025.10016)
Supplement: Aldoori et al. supplementary material [file S2632289725100169sup001.zip › O3FAs in vitro model paper supplementary table 1.docx]

**Supplementary Table 1. pH of *in vitro* fermentation reactions in the presence of omega-3 PUFAs and wheat bran**

|  | **baseline** | **8 hours** | **P^2^** | **24 hours** | **P^2^** |
| --- | --- | --- | --- | --- | --- |
| **no omega-3 PUFAs** | 7.45 (0.07)^1^ | 6.61 (0.23) | <0.001 | 6.36 (0.28) | <0.001 |
| **omega-3 PUFAs 1 μg/mL** | 7.46 (0.07) | 6.66 (0.22) | <0.001 | 6.40 (0.25) | <0.001 |
| **omega-3 PUFAs 25 μg/mL** | 7.46 (0.07) | 6.64 (0.23) | <0.001 | 6.37 (0.27) | <0.001 |
| **omega-3 PUFAs 50 μg/mL** | 7.45 (0.05) | 6.59 (0.21) | <0.001 | 6.37 (0.28) | <0.001 |

PUFAs, polyunsaturated fatty acids

^1^Mean (standard deviation) pH value for n=10 participants

^2^Paired t-test compared with the baseline value
