# Supplementary material for: The effect of omega-3 polyunsaturated fatty acids on short-chain fatty acid production and the gut microbiome in an in vitro colonic fermentation model
Source: Gut Microbiome (Camb). 2026 Jan 6;7:e1. doi: 10.1017/gmb.2025.10016 (PMC12835959; doi:10.1017/gmb.2025.10016)
Supplement: Aldoori et al. supplementary material [file S2632289725100169sup001.zip › O3FAs in vitro model paper supplementary table 2.docx]

**Supplementary Table 2. Short-chain fatty acid levels in *in vitro* fermentation reactions over time in the presence of omega-3 PUFAs and wheat bran**

| **Time** | **Experimental condition** | **acetate**  **(C2)^1^** | **propionate**  **(C3)** | **butyrate**  **(C4)** | **Total SCFAs** | **P^2^** | **% change from control^3^** | **P^2^** |
| --- | --- | --- | --- | --- | --- | --- | --- | --- |
| **8 hours** | **no omega-3 PUFAs** | 30.33 (6.16) | 8.56 (2.68) | 6.01 (1.88) | 44.95 (9.94) | - | - | - |
|  | **omega-3 PUFAs 1 μg/mL** | 30.82 (6.23) | 8.75 (2.27) | 6.05 (1.95) | 45.61 (8.09) | 0.69 | 2.81 (10.51) | 0.42 |
|  | **omega-3 PUFAs 25 μg/mL** | 30.90 (6.20) | 9.03 (2.40) | 6.05 (1.93) | 45.98 (8.21) | 0.54 | 3.68 (11.94) | 0.36 |
|  | **omega-3 PUFAs 50 μg/mL** | 29.27 (5.97) | 7.90 (3.56) | 5.90 (1.89) | 43.08 (8.95) | 0.23 | -3.63 (9.45) | 0.26 |
|  |  |  |  |  |  |  |  |  |
| **24 hours** | **no omega-3 PUFAs** | 39.37 (7.87) | 13.43 (2.99) | 10.76 (2.65) | 64.15 (9.85) | - | - | - |
|  | **omega-3 PUFAs 1 μg/mL** | 39.53 (8.47) | 13.13 (3.69) | 10.64 (2.87) | 63.31 (11.36) | 0.83 | -0.29 (15.96) | 0.95 |
|  | **omega-3 PUFAs 25 μg/mL** | 38.66 (8.03) | 13.27 (3.16) | 10.65 (2.48) | 62.58 (9.63) | 0.61 | -1.74 (12.23) | 0.66 |
|  | **omega-3 PUFAs 50 μg/mL** | 39.10 (8.03) | 14.42 (2.53) | 10.71 (2.69) | 64.22 (9.55) | 0.98 | 0.70 (10.58) | 0.84 |

C2, acetate; C3, propionate; C4, butyrate; PUFAs, polyunsaturated fatty acids; SCFAs, short-chain fatty acids

^1^mean (standard deviation) SCFA level (mmol/L) for n=10 participants

^2^Paired t-test comparing total SCFA level or % change with control (no omega-3 PUFAs)

^3^mean (standard deviation) % change of total SCFA level from the control (no omega-3 PUFAs) value
