# Supplementary material for: The effect of omega-3 polyunsaturated fatty acids on short-chain fatty acid production and the gut microbiome in an in vitro colonic fermentation model
Source: Gut Microbiome (Camb). 2026 Jan 6;7:e1. doi: 10.1017/gmb.2025.10016 (PMC12835959; doi:10.1017/gmb.2025.10016)
Supplement: Aldoori et al. supplementary material [file S2632289725100169sup001.zip › O3FAs in vitro model paper supplementary table 3.docx]

**Supplementary Table 3. pH of *in vitro* fermentation reactions in the presence of omega-3 PUFAs and pectin**

|  | **baseline** | **8 hours** | **P^2^** | **24 hours** | **P^2^** |
| --- | --- | --- | --- | --- | --- |
| **no omega-3 PUFAs** | 7.37 (0.13)^1^ | 6.24 (0.40) | <0.001 | 5.44 (0.18) | <0.001 |
| **omega-3 PUFAs 1 μg/mL** | 7.37 (0.09) | 6.33 (0.28) | <0.001 | 5.40 (0.19) | <0.001 |
| **omega-3 PUFAs 25 μg/mL** | 7.36 (0.09) | 6.24 (0.34) | <0.001 | 5.48 (0.19) | <0.001 |
| **omega-3 PUFAs 50 μg/mL** | 7.35 (0.08) | 6.23 (0.29) | <0.001 | 5.45 (0.27) | <0.001 |

PUFAs, polyunsaturated fatty acids

^1^Mean (standard deviation) pH value for n=9 participants (the omega-3 PUFA and pectin interaction was not tested in one participant due to the insufficient size of the faecal sample)

^2^Paired t-test compared with the baseline value
