# Supplementary material for: The effect of omega-3 polyunsaturated fatty acids on short-chain fatty acid production and the gut microbiome in an in vitro colonic fermentation model
Source: Gut Microbiome (Camb). 2026 Jan 6;7:e1. doi: 10.1017/gmb.2025.10016 (PMC12835959; doi:10.1017/gmb.2025.10016)
Supplement: Aldoori et al. supplementary material [file S2632289725100169sup001.zip › O3FAs in vitro model paper supplementary table 4.docx]

**Supplementary Table 4. Short-chain fatty acid levels in *in vitro* fermentation reactions over time in the presence of omega-3 PUFAs and pectin**

| **Time** | **Experimental condition** | **acetate**  **(C2)^1^** | **propionate**  **(C3)** | **butyrate**  **(C4)** | **Total SCFAs** | **P^2^** | **% change from control^3^** | **P^2^** |
| --- | --- | --- | --- | --- | --- | --- | --- | --- |
| **8 hours** | **no omega-3 PUFAs** | 39.12 (12.47) | 9.52 (4.55) | 5.27 (2.19) | 53.75 (17.00) | - | - | - |
|  | **omega-3 PUFAs 1 μg/mL** | 37.25 (12.61) | 8.94 (4.54) | 4.99 (2.08) | 51.19 (17.71) | 0.87 | 0.4 (22.7) | 0.96 |
|  | **omega-3 PUFAs 25 μg/mL** | 40.08 (15.17) | 9.11 (3.97) | 5.54 (2.52) | 54.62 (19.01) | 0.60 | 7.0 (27.6) | 0.47 |
|  | **omega-3 PUFAs 50 μg/mL** | 40.41 (12.23) | 9.97 (5.18) | 5.52 (2.35) | 55.90 (17.20) | 0.60 | 7.3 (22.6) | 0.09 |
|  |  |  |  |  |  |  |  |  |
| **24 hours** | **no omega-3 PUFAs** | 58.77 (10.49) | 16.03 (4.51) | 10.42 (2.37) | 83.37 (12.01) | - | - | - |
|  | **omega-3 PUFAs 1 μg/mL** | 61.27 (10.53) | 16.42 (4.13) | 10.48 (2.47) | 88.17 (11.77) | 0.004 | 6.4 (5.0) | 0.004 |
|  | **omega-3 PUFAs 25 μg/mL** | 57.75 (12.57) | 16.46 (5.60) | 10.56 (2.56) | 84.77 (13.71) | 0.66 | 2.7 (12.9) | 0.55 |
|  | **omega-3 PUFAs 50 μg/mL** | 58.63 (9.81) | 15.41 (4.81) | 10.74 (2.27) | 84.78 (11.58) | 0.31 | 1.9 (5.3) | 0.15 |

C2, acetate; C3, propionate; C4, butyrate; PUFAs, polyunsaturated fatty acids; SCFAs, short-chain fatty acids

^1^mean (standard deviation) SCFA level (mmol/L) for n=9 participants (the omega-3 PUFA and pectin interaction was not tested in one participant due to the insufficient size of the faecal sample)

^2^Paired t-test comparing total SCFA level or % change with control (no omega-3 PUFAs)

^3^mean (standard deviation) % change of total SCFA level from the control (no omega-3 PUFAs) value
